# Editorial: “Non-Coding RNAs in Head and Neck Squamous Cell Carcinoma”

**DOI:** 10.3389/fonc.2021.785001

**Published:** 2021-12-23

**Authors:** Wei Cao, Qiang Shen, Ming Yann Lim

**Affiliations:** ^1^ Department of Oral and Maxillofacial, Head and Neck Oncology, Shanghai Ninth People’s Hospital, Shanghai Jiao Tong University School of Medicine, Shanghai, China; ^2^ Shanghai Key Laboratory of Stomatology, Shanghai Research Institute of Stomatology, National Clinical Research Center of Stomatology, Shanghai, China; ^3^ Department of Genetics, Stanley S. Scott Cancer Center, School of Medicine, Louisiana State University Health Sciences Center, New Orleans, LA, United States; ^4^ Department of Otorhinolaryngology, Tan Tock Seng Hospital, Singapore, Singapore

**Keywords:** non-coding RNAs, microRNAs, circular RNAs, long non-coding RNAs, head and neck squamous cell carcinoma

Head and Neck Squamous Cell Carcinoma (HNSCC) is one of the most commonly reported malignant tumors in human beings, representing 5.3% of all cancers (1). In the past several years, there has been a lot of progress in revealing the mutational landscape of HNSCC. For example, a series of hotspot mutational genes such as *tumor protein p53* (TP53), *cyclin dependent kinase inhibitor 2A* (CDKN2A), *phosphatase and tensin homolog* (PTEN), *phosphatidylinositol-4,5-bisphosphate 3-kinase catalytic subunit alpha* (PIK3CA), and *notch receptor 1* (NOTCH1) were identified and tumors can be now classified based on genomic profiling (2, 3). In addition, epigenetic profiling revealed non-coding RNAs play a regulatory role in HNSCC (4, 5, Shao et al.). Non–coding RNAs including microRNAs (miRNAs), long non–coding RNAs (lncRNAs) and circular RNAs (circRNAs) have been found to be closely involved in the development and progression of HNSCC and partially associated with clinical significance of HNSCC patients.

## Part I: MicroRNAs and HNSCC

MicroRNAs are non–coding RNAs with about 22 nucleotides long that participate in the post–transcriptional regulation of gene expression involved in the proliferation, migration, metastasis and angiogenesis of cancer (6). For instant, microRNA–125a is responsible for the increased proliferation and migration of cancer cells by inhibiting the expression of the p53 protein (7). MicroRNA–363 promotes increased invasion and metastasis of HNSCC cells by targeting podoplanin protein (8). MicroRNA–300 influences epithelial to mesenchymal transition and metastasis by inhibiting the expression of the Twist (9). Paired box 4 (PAX4)–regulated microRNA–144/451 modulates invasion and metastasis by suppressing disintegrin and metalloproteinases (ADAMs) expression (10). MicroRNA–30e–5p inhibits angiogenesis and metastasis of cancer cells through directly targeting astrocyte elevated gene–1 (AEG–1) in HNSCC (11). In addition, a part of miRNAs derived from serum and salivary were found to be linked to the non–invasive diagnosis and prognosis of HNSCC patients. Serum exosomal microRNA–491–5p and microRNA–941 serve as the promising diagnostic biomarkers for HNSCC, respectively (12, 13). The three miRNA signature (microRNA–383, microRNA–615, and microRNA–877) or two–miRNA signature (miR–626 and miR–5100) act as the diagnostic and prognostic predictors for HNSCC patients, respectively (14, 15). Salivary miR–423–5p,miR–let–7a–5p,miR–3928 and a three–miRNA panel (miR–9, miR–134 and miR–191) could be used as novel non–invasive diagnostic biomarkers for HNSCC (16–18). More and more novel researches continue to strengthen the position of miRNAs as important regulator and useful biomarker in HNSCC as time goes on.

## Part II: Long Non–Coding RNAs and HNSCC

LncRNAs also belong to a subgroup of non–coding RNAs that are at least 200 nucleotides long and the majority of them do not have proteincoding ability (19). lncRNAs can be detected both in the nucleus and cytoplasm, and their different locations mean different functions that were involved in regulation of gene expression, e.g., chromatin modification, interaction with transcriptional factors, mRNA processing, cell metabolism, proliferation, apoptosis, acting as “molecular sponge” and creating ribonucleoprotein complexes (19–22). For example, Metastasis–associated lung adenocarcinoma transcript 1 (MALAT1) has one of the most conserved primary and secondary structures of all lncRNAs (23). Knockdown of MALAT–1 led to impaired migration and proliferation ability *in vitro* and fewer metastases *in vivo* in HNSCC cells (24). MALAT–1 transcriptionally activated by signal transducer and activator of transcription 3 (STAT3) induces epithelial–to–mesenchymal transition (EMT) and accelerates HNSCC metastasis by interacting reciprocally with miR–30a (25). HOX transcript antisense RNA (HOTAIR) is expressed from locus *homeobox C cluster* (HOXC) and lncRNA HOTAIR transcriptionally activated by STAT3 interact with pEZH2–S21 resulting in proliferation and the growth of HNSCC xenograft tumors *in vivo* (26). Besides, some of lncRNAs functionally act as tumor suppressors, e.g., lincRNA–p21 (27) or LINC02487(Feng et al.), long non–coding RNA Fer–1–like protein 4 (FER1L4) (28), and growth arrest specific 5 (GAS5) (29), but others play an oncogenic role, e.g., ring finger and CCCH–type domains 2 (RC3H2) (30), FOXD2 adjacent opposite strand RNA 1(FOXD2–AS1) (31), KTN1 antisense RNA 1(KTN1–AS1) (32), LINC00460 (33), H19 imprinted maternally expressed transcript (H19) (34), small nucleolar RNA host gene 6 (SNHG6) (35), urothelial cancer associated 1 (UCA1) (36), or ZNFX1 antisense RNA 1 (ZFAS1) (37), and can be used as therapeutic targets in the future. More and more evidences show that multiple lncRNAs–based signatures could be diagnostic and prognostic predictors of HNSCC patients by mathematical modeling. A nomogram based on an 8–lncRNA signature was identified as a novel diagnostic biomarker for HNSCC (38). A three–lncRNA signature was screened and identified to well predict the survival of HNSCC patients (39). Immune–related and autophagy–related lncRNA signatures were respectively developed into prognostic indicator for HNSCC (40, Guo et al).

## Part III: Circular RNAs and HNSCC

circRNAs are a novel subclass of non–coding RNAs, which are produced by reverse splicing and are characterized by a closed single–stranded structure and lack of 5′cap and 3′polyadenylation [poly(A)] tail (41). Currently, the most established function of circRNAs is that circRNAs can act as miRNA sponges resulting in the initiation and progression of cancer and play a regulatory role in the tumor microenvironment (TME) (42, 43). For example, circRNA Pvt1 oncogene (circPVT1) transcriptionally enhanced by the mut–p53/Yes1 associated transcriptional regulator (YAP)/TEAD complex promotes the proliferation of HNSCC cells by modulating the expression of miR–497–5p (44). CircRNA_ 036186 likely regulates 14–3–3ζ expression by functioning as a ceRNA in the development and progression of HNSCC (45). And the function as a ceRNA was also found to be in circRNA_100533 (46), circRNA myosin light chain kinase (circMYLK) (47), circRNA_0042666 (48), circRNA_100290 (49), circRNA_0000140 (50), circRNA Matrin 3(circMATR3) (51), circRNA_0000495 (52),circRNA coronin 1C (circCORO1C) (53), circRNA_0036722 (54), circRNA_0000218 (55), circRNA epithelial stromal interaction 1(circEPSTI1) (56), circRNA_103862 (57), circRNA_009755 (58), circRNA septin 9 (circSEPT9) (59), circRNA par–3 family cell polarity regulator (circPARD3) (60), circRNA BICD cargo adaptor 2 (circBICD2) (61), circKIAA0907 (62), circRNA_0023028 (63), circRNA_0000700 (64), circRNA ATP binding cassette subfamily B member 10 (circABCB10) (65), circRNA_0042823 (66), circBCL11B (67), circRNA fibronectin type III domain containing 3B (circFNDC3B) (68), and circRNA PTPRF interacting protein alpha 1(circPPFIA1) (69). In addition, a few circRNAs can directly interact with proteins and enzymes as protein scaffolds. For instance, circRNA G protein subunit gamma 7 (circGNG7) inhibits HNSCC cell proliferation by binding to serine residues 78 and 82 of Heat shock protein 27 (HSP27) (70). CircRNA FAT atypical cadherin 1(circFAT1) prevents STAT3 dephosphorylation by binding to STAT3, resulting in HNSCC stemness and immune evasion (71). Due to more stable than lncRNA and miRNA, circRNAs are regarded as an ideal diagnostic predictor tool in clinical practice. In HNSCC, circRNA_0003829 and circRNA_0036722 serve as diagnostic predictors with AUC=0.81 and AUC=0.83, respectively (54, 72). Plasma–derived the three circRNA panel well predicts the occurrence of HNSCC (73).

This Research Topic *“Non–coding RNAs in Head and Neck Squamous Cell Carcinoma: Functional and Clinical Implications”* includes 21 original articles and 3 review articles, which highlight the regulatory mechanism and clinical relevance of non–coding RNAs in HNSCC. For example, Wan et al. identifies a super–enhancer regulatory model of miR–21–5p by FOS like 1 (FOSL1), promoting malignant progression of HNSCC. The article by Li et al. discuss LINC02195 as a regulator of MHC I and the number of CD8^+^ and CD4^+^ T cells in the tumor microenvironment. Moreover, Wang et al. summarizes the crosstalk between lncRNAs and microRNAs as well as the detailed regulatory mechanism of the interaction. Additionally, Luo et al. identifies miRNAs signatures respectively for HPV+ and HPV− HNSCC, which are of great significance in evaluating patient survival. All of other articles mainly discuss the potential function of non–coding RNA as a ceRNA in HNSCC.

## Author Contributions

All authors listed have made a substantial, direct and intellectual contribution to the work, and approved it for publication.

## Funding

WC was supported by grants from the National Natural Science Foundation of China (81972589).

## Conflict of Interest

The authors declare that the research was conducted in the absence of any commercial or financial relationships that could be construed as a potential conflict of interest.

## Publisher’s Note

All claims expressed in this article are solely those of the authors and do not necessarily represent those of their affiliated organizations, or those of the publisher, the editors and the reviewers. Any product that may be evaluated in this article, or claim that may be made by its manufacturer, is not guaranteed or endorsed by the publisher.
